# Exploring COVID-19 Vaccine Attitudes among Racially and Ethnically Minoritized Communities: Community Partners’ and Residents’ Perspectives

**DOI:** 10.3390/ijerph20043372

**Published:** 2023-02-14

**Authors:** Isabel Martinez Leal, Journa Njoh, Tzuan A. Chen, Faith Foreman-Hays, Brian C. Reed, Sean A. Haley, Kerry Chavez, Lorraine R. Reitzel, Ezemenari M. Obasi

**Affiliations:** 1 Department of Health Disparities Research, MD Anderson Cancer Center, Houston, TX 77230, USA; 2 Department of Psychological, Health & Learning Sciences, The University of Houston, 3657 Cullen Blvd., Stephen Power Farish Hall, Houston, TX 77204, USA; 3 HEALTH Research Institute, The University of Houston, 4349 Martin Luther King Blvd., Houston, TX 77204, USA; 4 Houston Health Department, 8000 North Stadium Dr., Houston, TX 77054, USA; 5 Department of Clinical Sciences, Tillman J. Fertitta Family College of Medicine, The University of Houston, 5055 Medical Circle, Houston, TX 77204, USA; 6 Center for Civic & Public Policy Improvement, 5445 Almeda Rd., Suite 504, Houston, TX 77004, USA

**Keywords:** health equity, black, indigenous, People of Color (BIPOC) communities, COVID-19 vaccination, qualitative research

## Abstract

COVID-19 has disproportionately affected Black, Indigenous, and People of Color (BIPOC) communities, yet rates of COVID-19 vaccination remain low among these groups. A qualitative study was undertaken to better understand the factors contributing to low vaccine acceptance among these communities. Seventeen focus groups were conducted in English and Spanish from 8/21 to 9/22, with representatives from five critical community sectors: (1) public health departments (*n* = 1); (2) Federally Qualified Health Centers (*n* = 2); (3) community-based organizations (*n* = 1); (4) faith-based organizations (*n* = 2); and (5) BIPOC residents in six high-risk, underserved communities in metropolitan Houston (*n* = 11), for a total of 79 participants, comprising 22 community partners and 57 community residents. A social-ecological model and anti-racism framework were adopted to guide data analysis using thematic analysis and constant comparison, which yielded five key themes: (1) legacy of structural racism: distrust and threat; (2) media misinformation: mass and social; (3) listening and adapting to community needs; (4) evolving attitudes towards vaccination; and (5) understanding alternative health belief systems. Although structural racism was a key driver of vaccine uptake, a notable finding indicated community residents’ vaccine attitudes can be changed once they are confident of the protective benefits of vaccination. Study recommendations include adopting an explicitly anti-racist lens to: (1) listen to community members’ needs and concerns, acknowledge their justified institutional distrust concerning vaccines, and learn community members’ healthcare priorities to inform initiatives built on local data; (2) address misinformation via culturally informed, consistent messaging tailored to communal concerns and delivered by trusted local leaders through multimodal community forums; (3) take vaccines to where people live through pop-up clinics, churches, and community centers for distribution via trusted community members, with educational campaigns tailored to the needs of distinct communities; (4) establish vaccine equity task forces to continue developing sustainable policies, structures, programs and practices to address the structural issues driving vaccine and health inequities within BIPOC communities; and (5) continue investing in an effective infrastructure for healthcare education and delivery, essential for competently responding to the ongoing healthcare and other emergency crises that impact BIPOC communities to achieve racial justice and health equity in the US. Findings underscore the crucial need to provide culturally tailored health education and vaccination initiatives, focused on cultural humility, bidirectionality, and mutual respect to support vaccine re-evaluation.

## 1. Introduction

SARS-CoV-2 infections, hospitalization, and mortality have disproportionately impacted Black, Indigenous, and People of Color (BIPOC) communities throughout the United States (US) [1,2,3,4,5,6,7]. According to recent data from the Centers for Disease Control and Prevention (CDC), Black Americans are 1.7 times more likely to die and 2.5 times more likely to be hospitalized from COVID-19 than White Americans [8]. Vaccine uptake disparities have been concerning across BIPOC communities, despite the rollout of Pfizer, Moderna, and Johnson & Johnson vaccines and their high effectiveness in preventing severe SARS-CoV-2 infection-related illnesses and death [9,10,11,12,13]. In April 2021, according to the CDC, the rates of adults who reported having received at least one dose of the COVID-19 vaccine were higher among Asian and non-Hispanic White adults (69.6% and 59.0%, respectively), with lower rates observed among Hispanic (47.3%), non-Hispanic Black (46.3%), Native Hawaiian or other Pacific Islander (NH/OPI) (45.9%), American Indian or Alaskan Native (AI/AN) (38.7%), and adults of multiple or other race (42.6%) [14]. These patterns were also observed in Texas according to an online survey of Texans aged 18 years and older, collected between December 2021 and March 2022, wherein higher rates of unvaccinated individuals were found among minoritized communities; 39% of Black and 40% of Hispanic adults reported being unvaccinated compared to 32.5% of White adults [15]. As of November 2021, however, CDC survey data has indicated that vaccine uptake disparities have since narrowed [14], with 72.1% Asian, 56.2% non-Hispanic White, 66.2% Hispanic, 50.5% non-Hispanic Black, 70.5% NH/OPI and 77.2% AI/AN adults reporting having received at least one dose of the COVID-19 vaccine [16]. Despite higher vaccination rates in the larger general US population, vaccine uptake remains a concern among racial and ethnic groups, with continuing disparities in the Midwest and urban areas [14,17]. Additionally, Black Americans only make up 10% of those who have recently received at least one dose of the vaccine, relative to their share of the population (12%), while this gap has closed for Latino/a/x adults, who comprise a larger share (21%) of those recently vaccinated [18].

To increase vaccination uptake within minoritized communities and prevent premature deaths from COVID-19, more information about what underlies the lower vaccination rates is needed. Analysis of behavioral and social drivers of COVID-19 vaccine attitudes and uncertainty (concerns about contracting COVID-19, safety and importance of vaccine, social norms regarding vaccines, healthcare provider vaccine recommendation, and access to vaccine) among unvaccinated racial and ethnic minoritized individuals in the US revealed that large proportions of AI/AN, Black, and multiple and other race adults chose to remain unvaccinated despite acknowledging the importance and safety of COVID-19 vaccines [14]. Vaccine uptake also remains a concern among those fully vaccinated as disparities in booster dose vaccination uptake emerge [14,15]. The same trend has been observed in Texas, with higher rates of vaccine booster uncertainty expressed by Black (22.5%), Hispanic (17.8%), and multiple and other race (19.7%) adults compared to White adults (16.2%) [15]. These data suggest the urgent need to understand the factors affecting COVID-19 vaccine attitudes, access, and uncertainty among minoritized communities.

Constructs like vaccine “hesitancy” are often misrepresented and limited in their ability to explain lower vaccination rates in BIPOC communities, minimizing the significant effects of structural racism and the social determinants of health and related inequities (SDOHRI), which are non-medical causal factors impacting health [19] (e.g., access to quality healthcare, built environment, social inclusion and non-discrimination, education, employment, language/literacy, socioeconomic conditions, and transportation) on COVID-19 vaccine access and uptake [10,20,21,22,23,24,25]. While the term vaccine “hesitancy” has commonly been used in research literature, the framing of “hesitancy” among BIPOC communities to be vaccinated can mistakenly place the onus for vaccination on these community members to become less “hesitant”, rather than on medical and public health systems to become more trustworthy [26]. “Hesitant,” from the Latin haesitans, means “to stick fast, left undecided [27],” which denotes waiting or a passive state. The term “deliberation” proposed by Corbie-Smith is used herein instead, as it denotes an active state: “to consider, weigh well [28].” This term more accurately conveys community members’ reasonable and active questioning and evaluation of vaccine efficacy and the medical and governmental systems that have been the source of historical and ongoing racially/ethnically based ethical violations, abuses of power, discrimination, and racism [29]. Adopting a stance of actively questioning COVID-19 vaccination by BIPOC community members is sensible, as well as a means of claiming agency among those for whom power has been and continues to be denied.

Structural racism is defined as the totality of societal structures and policies that create and uphold harmful social conditions, poverty, and other facets of social disadvantage and inequity by unequally disseminating access to opportunities and societal resources by race and ethnicity [30,31]. These structural inequities negatively impact SDOHRI and have rendered BIPOC communities vulnerable to the SARS-CoV-2 virus [10,21,22,23,24,25,31,32,33,34]. Extant literature offers evidence suggesting that unwillingness to receive vaccination may be deeply influenced by a general mistrust of health authorities. There is an extensive history of earned medical mistrust among BIPOC communities in the US. The mere mention of the infamous Tuskegee Syphilis Study and the exploitation of Henrietta Lacks’ biospecimen oversimplifies and minimizes the depth of the BIPOC experience and the impact that structural racism has had on the consequent distrust of healthcare [21,22,23,24,25]. These historical and ongoing experiences of harmful treatment of BIPOC community members have established reservations concerning the trustworthiness of science, research institutions, healthcare systems, and the government [26,29,30]). This distrust also extends to perceptions of vaccine safety and effectiveness [21,24,35]. The consequences of lived experiences of structural racism (e.g., engagement with the healthcare system), coupled with mixed political messaging (e.g., bleach, hydroxychloroquine) [36], risk perception of COVID-19 [37], perceived vaccine development process (e.g., Operation Warp Speed) [10], and the SDOH [22,23] significantly affect COVID-19 vaccine beliefs and consequently access and uptake. Despite the fact that city and county systems may be called upon to protect the health of their community members through vaccination drives, little work has examined how local contexts may also influence vaccine uptake. Therefore, there is an urgent need to further explore potential barriers to vaccine acceptance within discrete geographical locations to ensure that subsequent outreach efforts address the most critical factors for vaccine receipt, particularly within communities with lower uptake rates.

The present study explored the various experiences of BIPOC community members to understand the factors affecting COVID-19 vaccine attitudes, access, and acceptance. While several models attempt to explain and mitigate vaccine uncertainty [38], other than the model developed by Abdul-Mutakabbir (2022), few address the BIPOC experience and go beyond myth corrections [39,40]. The exclusion of BIPOC populations as investigators, participants, and informants of research significantly contributes to the generation and maintenance of health disparities, including COVID-19 vaccine uptake, as less is known about factors affecting health among underrepresented groups and how to address them using culturally informed methods [41]. The present study emphasizes community ownership in a COVID-19 public health study by exploring local BIPOC community members’ lived experiences of the pandemic. Both community partners involved in vaccine distribution and outreach and community residents’ experiences are explored, providing insights into the various challenges and successes partners faced with vaccination efforts, as well as community residents’ vaccination perspectives, experiences, and deliberations and the barriers and facilitators to vaccine access. Combining these distinct perspectives helps to fill a gap in the literature by providing a more complete and inclusive view of the challenges and concerns confronting BIPOC communities in addressing COVID-19 vaccination to inform the development of a community-centered and culturally responsive vaccination program focused on health equity. This study thus places communities of color at the core of the research process, as this is essential for understanding and addressing COVID-19 vaccine deliberation that affects vaccine uptake and sustainment for boosters in BIPOC communities [10]. Study findings inform recommendations on the importance of: listening to and including community members in the development of vaccine campaigns culturally tailored to the needs of diverse communities that are based on cultural humility, bidirectionality, and acknowledgement and respect for BIPOC community members’ justified institutional distrust; and continued investment in an effective infrastructure for healthcare education and delivery within these under-resourced communities capable of responding to their ongoing healthcare crises and necessary to advance racial justice and health equity in the US.

## 2. Materials and Methods

### 2.1. Study Design

The present study reports on the findings from the exploratory qualitative component of a mixed-method project based on semi-structured focus groups. The parent study focused on investigating and developing a comprehensive framework to understand the multilevel and multidomain factors affecting COVID-19 vaccine attitudes, access, and series completion among underserved BIPOC communities in Houston, Texas. Findings from this convergent mixed-method project will be used to inform the development and piloting of a community-centered, culturally responsive COVID-19 vaccine implementation program. A social constructionist approach was adopted, as this approach best aligned with the research aim guiding the qualitative component of understanding how participants construct their experiences and perceptions of COVID-19 and vaccination. Social constructionism is a theory that challenges taken-for-granted assumptions about the world as unitary, fixed, and inherently existent; adopting the view that individuals construct themselves, others, and the world through their social interactions, and as such, acknowledges the existence of multiple realities, truths, and selves [42]. Given that structural racism rests upon mutually reinforcing systems of inequity that together produce racial health inequities [30], we also drew upon an anti-racism framework [31] and the social-ecological model [43] to understand the multilevel factors affecting participants’ attitudes and behaviors towards COVID-19 vaccine uptake. The Internal Review Board of the University of Houston approved all study procedures for protecting human subjects.

### 2.2. Participants 

For focus group participation, researchers recruited five critical community sector representatives, who were ≤18 years old, spoke English or Spanish, and either served or were from underserved BIPOC—in our sample mostly Black and Latino/a/x—communities representing: (1) public health departments; (2) community-based organizations; (3) Federally Qualified Health Centers (FQHCs); (4) faith-based organizations; and (5) BIPOC residents in six high-risk, underserved communities in metropolitan Houston. Participants were compensated with a USD 50 electronic gift card for attending a focus group.

### 2.3. Recruitment and Consent

Phase 1 recruitment of community partners lasted from August to September 2021. In Phase 2, community residents were recruited from among English- and Spanish-speaking communities between April and September 2022. Stratified purposive sampling was used to select participants. As initial recruitment efforts yielded a high number of vaccinated community residents, further efforts continued to focus on purposively recruiting unvaccinated participants. As a community-centered project, recruitment of focus group participants was undertaken by community education and outreach coordinators and community health workers of the Community Engagement Core of the HEALTH Center for Addictions Research and Cancer Prevention, funded by the National Institute of Health and National Institute on Minority Health and Health Disparities’ Research Centers in Minority Institutes (RCMI U54MD015946) at the University of Houston. Recruitment and other study procedures were facilitated by the Community Advisory Board (CAB), the Community Research Advisory Board (CRAB), and strong long-standing [44] community relationships [39].

During the consent process, the research team discussed the nature of the study and focus groups with all participants, who provided written consent prior to participation. Participants were informed that, given the shared nature of group interviews, the research team could not ensure privacy, and asked participants to respect each other’s privacy during the focus group. However, to safeguard confidentiality, researchers informed participants that any identifying information would be stripped from the focus group transcripts, only deidentified data would be retained and only pseudonyms would be used in any reporting of findings. According to whether the focus group was conducted in-person or virtually, permission for audio-recording was solicited and granted by all in-person participants, and for audio and video recording for all virtual participants.

### 2.4. Data Collection

Focus groups were conducted using semi-structured focus group guides from August 2021 to September 2022, in English or Spanish, according to participant preference, by a bilingual cultural anthropologist and public health researcher (IML) trained in qualitative methods. Interview guides were developed in accordance with research aims and were field tested and refined based on participant responses in the field [45]. The semi-structured focus group guides were tailored for representatives from each of the different community member groups, i.e., public health departments, FQHCs, community organizations, faith-based organizations, and community residents. The RCMI CRAB reviewed, provided feedback on, and approved each focus group guide. As this research was conducted during the COVID-19 pandemic, focus groups occurring in 2021 took place virtually using a videoconferencing platform (Zoom) and were audio- and video-recorded. Later focus groups conducted in 2022 were mostly in-person at local community centers and churches and were audio-recorded. Focus groups consisted of 3–10 participants and lasted 60–90 min. For community partners serving BIPOC communities, interview questions focused on: guidelines used to inform COVID-19 efforts and vaccine distribution, outside authorities influencing organizations’ pandemic prevention efforts, perceived main barriers to uptake of vaccines (e.g., accessibility, attitudes), personal concerns about the vaccine, satisfaction with current city-wide vaccine drives to community members, organizational barriers experienced in vaccine provision, efforts made to achieve equity in vaccine distribution, provision of education culturally tailored to BIPOC communities to address medical mistrust and structural racism, and support needed to facilitate vaccine uptake and distribution. Questions for community residents included: knowledge about COVID-19, vaccines, and information sources; perceived personal and communal threat of the virus; attitudes and experiences in following safety precautions against viral contagion; personal COVID-19 experience (e.g., family, friends, employment) and vaccine concerns; importance of being vaccinated against the virus; what was needed to feel comfortable with receiving the vaccine; any perceived barriers to being vaccinated; changes in attitudes towards being vaccinated; and suggestions for improving prevention of COVID-19 for individuals personally as well as communally.

### 2.5. Data Analysis

Descriptive statistics, including mean and standard deviation (SD) and percent, were provided for continuous and categorical variables of interest, respectively. Quantitative data were analyzed using SAS 9.4 (SAS Institute, Cary, NC, USA). Focus groups were recorded and transcribed verbatim by a professional transcription service and uploaded onto ATLAS.ti 9 (ATLAS.ti, Scientific Software Development, version 9.1.6, Berlin, Germany, 2020) to organize data analysis. Recordings of focus groups conducted in Spanish were also professionally translated and transcribed into English to facilitate team coding. Accuracy of English translation was verified by the bilingual first author (IML). Thematic analysis and constant comparison were used to systematically code, categorize, and identify themes within the data [46]. Coding entailed an iterative process using constant comparison, wherein new transcripts were compared within and across previously coded transcripts to combine and condense codes into categories and themes drawn from the data, rather than being predetermined. The social-ecological model and anti-racism framework framed data analysis, guiding the categorization of inductive codes into meaningful themes in an interactive, deductive, and inductive process of interpretation [47]. The first two authors (IML) and (JN), both trained in qualitative analysis, independently coded five transcripts to develop an initial coding frame and met to discuss and resolve any discrepancies in coding and to refine the coding frame. Throughout the data analysis process, the coding frame remained open to refinement and development of new themes. The two analysts met to discuss and agree upon a final frame that was reapplied to all the data. The constant comparison process served to refine codes, avoid redundancy, confirm accurate reporting of all the data, support development of appropriate categories and themes, and achieve data saturation, i.e., the point in data analysis when no new information is found, signaling the end of data collection [48].

## 3. Results

### 3.1. Sample Characteristics

Overall, 17 focus groups were conducted with 79 participants in total, representing 22 stakeholders from public and community health organizations and faith-based organizations and 57 community residents. Focus groups conducted in English included one with five public health department representatives; two with FQHCs consisting of five representatives; one with six representatives from different community organizations; one with four different faith-based organizations; and nine with 43 community residents representing six different BIPOC communities. Spanish focus groups consisted of one with two representatives of different faith-based organizations, and two focus groups with 13 community residents representing two different BIPOC communities. Key demographic characteristics of focus group participants in Phases 1 and 2 were collected through online surveys, along with vaccination status (Table 1). However, as participants could opt out of responses yet continue with surveys, there are missing data in Table 1. Response rates were 86.36% among community partners and 63.16% among community residents. Of the 79 focus group participants, one participated in both the community partner and community resident focus groups, but only completed one survey, and there were 23 individuals who did not complete the survey. Of these 23, 11 were elderly individuals who did not have email addresses; therefore, they were not sent survey invites, and 11 adults failed to submit their survey responses, leaving 55 survey responses. Phase 1 partners self-reported higher total household income and higher educational level and were also more likely to be employed, to be covered by private insurance/military healthcare, and to have received at least one dose of the COVID-19 vaccine (Table 1).

### 3.2. Themes

In keeping with the social-ecological framework that informed data analysis, our findings point to the dynamic interaction of multidimensional factors shaping vaccination uncertainty across distinct levels of influence. As factors interact across levels, they are not discrete.

**Figure 1:**
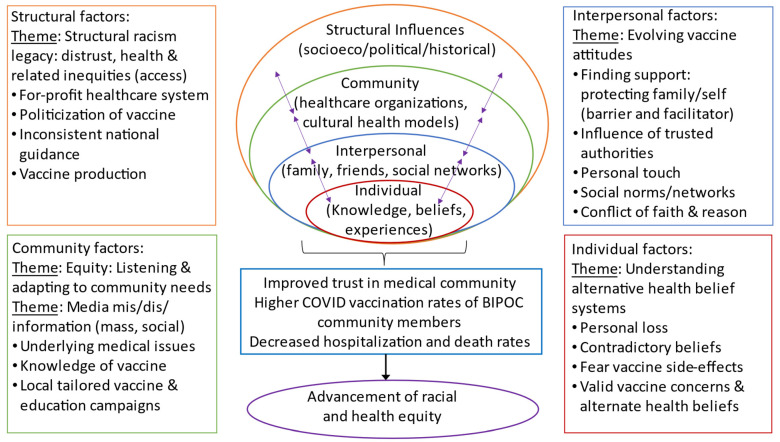
Interactions of Factors Affecting Vaccine Deliberation among BIPOC Communities.

We identified five main themes that are further delineated into the specific categories (Figure 1): (1) legacy of structural racism: distrust and threat (structural level); (2) media misinformation: mass and social (structural and community levels); (3) equity: listening and adapting to community needs (community level); (4) evolving attitudes towards vaccination (community and interpersonal level); and (5) understanding alternative health belief systems (all levels). These themes are related to issues of uncertainty, access, and alternative health belief systems.

#### 3.2.1. Structural Factors—Legacy of Structural Racism: Distrust and Threat

Structural inequities were identified by participants as critical factors underlying vaccine uncertainty and access. Among community partners and residents alike within Black and Latino/a/x communities, the issue of institutional and medical distrust was reported as a critical factor in choosing not to be vaccinated. (The term “distrust” is used here instead of the more commonly used “mistrust,” as distrust conveys the meaning of “express[ing] a lack of trust stemming from a specific experience or certain knowledge” (https://www.dictionary.com/e/mistrust-vs-distrust/, accessed 16 November 2022), i.e., based on prior experience, which aligns with our intent of communicating that medical distrust among BIPOC community members is earned due to historical exploitation and brutality.) Additionally, the development of the COVID-19 vaccines under the Emergency Use Authorization by the Food and Drug Administration, granting expedited vaccine production and testing, only served to augment safety concerns among many participants. For Latino/a/x populations, this also included concerns around being undocumented. Several Black American and Latino/a/x community residents also did not have medical insurance, limiting the healthcare they could access. Community partners noted that COVID-19 arose at a time when institutional distrust was intensified by the routine killing of Black Americans by police, who were regularly exonerated for these crimes, and the political activism of the Black Lives Matter movement to address this brutality, increasing institutional distrust among BIPOC communities:

*The virus comes in a time where we are having a hard time trusting the government because we were starting to catch all these videos […] obvious to the people living in that area and that were witnesses to these crimes and police brutality, but they have put it under the rug for so long that people have had a hard time trusting local government and officials. And so, now you have this whole COVID thing going on and now people are like, “Well, I’m not too sure. This might be like a government kind of thing.” And so, I think that has a lot to do with why individuals have a really hard time trusting the government.*

(Community partner, FQHC)

Participants were aware of the historical medical exploitation of Black people in the US by the government and academic researchers, and were justifiably distrustful of intense vaccination campaign efforts targeting BIPOC communities:

*I think something that startled or made people uneasy about the vaccine is that it’s free. America, at the very least, nothing in healthcare is free. If something is free in healthcare, it’s probably bad or lower quality. And don’t forget, people are afraid of the federal government, Tuskegee […] it was always in the poor neighborhoods where they were trying to get people to take the shot-you didn’t really see them pushing it in the upper echelon classes. And it just makes the people in the neighborhood wonder, “What are you trying to give us? What is it you’re not telling me?”*

(Community resident, 3rd Ward)

Black participants especially stressed that, given the persistent degradation and hardship of structural racism experienced by community residents—e.g., still recovering from having lost their homes during Hurricane Harvey in 2017, for which compensation from the government had yet to be received—many felt overwhelmed by the multiple, intersecting disadvantages, i.e., unemployment, food insecurity, unstable housing, limited access to healthcare, because of the for-profit healthcare system, that were all being compounded by COVID-19, sending these communities into survival mode:

*People are not worried about being vaccinated again now, people are mostly worried about, “How am I going to live tomorrow? Where are me and my children going to stay if we can’t pay our rent still?” We have a lack of food and lack of resources. They’re not trying to run and go get vaccinated right now, I think the vaccination, even though we have people dying every day from the Delta variant, that might be, like, number three or four on their list when they’re trying to survive. We’re in sur–they’re in survival mode, that’s where I believe we’re at.*

(Community partner, community organization)

These intersecting vulnerabilities only made community residents feel more unsafe at a time when the safety of Black Americans was being highlighted in the media through the political activism of Black Lives Matter:

*We [Black Americans] don’t trust. We will never trust people, practically, around us because we cannot trust other faces. And I hate to say it like that, I know that it has to be said, we innately […] we want to, but we know that that face can turn on us… So, we’re not in safe places. From the moment we wake up and leave our homes to the moment we come back to our homes, we’re not in safe spaces.*

(Community partner, community organization)

#### 3.2.2. Structural and Community Factors—Media Misinformation: Mass and Social 

Media misinformation has been an important factor influencing vaccination uptake that has a multidimensional effect across levels—structural, community, interpersonal and individual—along with the related influence of vaccine politicization. Given the long-standing structural inequities experienced by BIPOC communities, many community residents distrusted mainstream communications regarding COVID-19 based on earned medical distrust and may not have obtained the educational resources required to understand these medical communications. While community members’ questioning the safety and efficacy of the COVID-19 vaccines itself springs from structural inequities and power differentials between White privileged and BIPOC communities, this left these members vulnerable to the massive mis/disinformation campaigns on vaccine safety. Community partners and residents both stressed the need to directly acknowledge and correct vaccine disinformation within BIPOC communities:

*Now there’s still going to be disinformation…that’s gonna keep people from getting vaccinated and I think those are the people that we just have to continue to work on because they’re getting worked by the social media, by brothers, sisters, sister-in-law’s friend, and so we just have to be equally as persistent to share with them the information and the benefits of getting vaccinated […] to say to my own patients, “I was vaccinated. My children were vaccinated. My husband was vaccinated, and we all understand what you’re saying around why you’re hesitant, the things that have happened in the past that have been pretty horrific to our community, but let us not be left behind, because we are the ones that are being hospitalized. We are the ones in the ICU. So, make sure that you’re–that we take good care of ourselves and part of that is to get vaccinated. I’ve been vaccinated. It’s safe. It’s okay.”*

(Community partner, FQHC)

Community residents also spoke to the prevalence of disinformation and how they sought to correct these mistaken views on the vaccine among neighbors:

*When you walk through the neighborhood as far as Blacks go, you hear the fact that it’s a government conspiracy, that they’ve designed this thing to hit Blacks, then you have Black folks out there that don’t believe this actually exists at all. When I say exist, it’s non-existent and that it’s designed to basically get rid of us if it does exist. And you go round and round trying to explain it to people to get them to understand.*

(Community resident, 3rd Ward)

Additionally, due to the confused, contradictory information on COVID-19 released by the medical community, as well as by the US government—particularly during the Trump administration, whose actions downplayed the threat of COVID-19 and whose messaging about the dangers of the pandemic disputed those of the scientific community—community residents did not know who to believe and called for consistent, coordinated information and messaging:

*I would say when you put information out, put it out there correctly the first time. So, you don’t have to go back and adjust what you said, creating chaos and confusion because I think there’s been some inconsistent messages…It [COVID-19] could’ve been prevented...we didn’t move in a timely manner in terms of researching, we didn’t get the information in time, the Trump administration dismissed that group of researchers. If the Trump administration had acted a lot sooner, it’d been less deaths.*

(Community resident, 5th Ward)

Participants explicitly addressed the politicization of vaccination as contributing to vaccine disinformation, inconsistency in messaging, and creation of confusion and discord; some perceived it as the primary driver of vaccine uncertainty:

*Moderator: If you could tell city, state, or national leadership one thing that would improve the prevention of COVID-19 for you and your community, what would it be?*

*Respondent: I don’t know if this is kosher or not, but I would tell them to leave public health out of politics. [Three other participants agree]…the communication’s been very inconsistent and causes inherent mistrust.*

(Community partner, Health Dept.)

Community residents also expressed frustration at the politicizing of the COVID-19 vaccine and questioned the motivation for doing so:

*What in the world made this vaccination so politicized? I think it had a tremendous negative effect on people taking that vaccine because they politicized it. I was hard-pressed to understand why would they make a political issue out of a health issue? I was very hard-pressed to wrap that around my brain. This is not about politics. This is about the health of not only this country, but the health of the world. Why would you politicize that?*

(Community resident, 5th Ward)

#### 3.2.3. Community Factors—Listening and Adapting to Community Needs 

At various points throughout the focus groups, participants stressed the need to engage community residents and leaders and to have their voices and experiences guide outreach efforts, rather than adopting a paternalistic attitude of being outside experts that know what community residents need:

*Having that equity lens on everything we do so that we’re meeting the community where they are with culturally representative individuals as our community health workers, they speak the same language of the community they’re going into and have the same type of experiences and are able to really communicate on that same level. So that we’re not talking at somebody, we’re talking as part of the community, as someone that has gone through the same hesitancy or struggle or understands the access issues and can kinda really relate and talk on that level instead of prescribing “This is what you should do.”*

(Community partner, Health Dept.)

Likewise, community residents as well as partners were aware of the importance of developing COVID-19 vaccination efforts that are culturally informed and adapted to individual communities as part of the effort to increase access to vaccines:

*I would tell him to have more outreach workers, let’s say, that would go into these community centers and explain exactly what things are in whatever language it has to be given, be that Vietnamese, Spanish, Urdu, and Indian, whatever, as long as the message is given out to reach different cultures.*

(Community resident, Gulfton)

Participants from both groups noted that community residents often reported having chronic underlying health conditions, such as diabetes or hypertension, known to be inequitably distributed in BIPOC communities, and had concerns about whether to be vaccinated given these conditions. Underlying conditions could motivate community residents to be vaccinated:

*Then we have our parents who did it [were vaccinated] because of their own comorbidities, right? “I know I have diabetes. I just had a transplant. My wife is going to the cancer chemotherapy.” People are protecting themselves and their family members. They understand, “I really need to protect myself.”*

(Community partner, FQHC)

Conversely, concerns about chronic conditions could lead people not to get vaccinated:

*I agree with her because I have sickle cell, so I’m not putting all that extra medicine in there and I don’t know what it is.*

(Community resident, Greenspoint 2)

Participants working in public health departments as well as physicians with FQHCs reported that, while healthcare organizations had made a concerted and effective effort to address structural inequities, particularly regarding community outreach educational and vaccination programs, the infrastructure to support these efforts had initially been lacking. Notably, they reported the need to integrate health education and community outreach provision into routine practice built into their infrastructure:

*It’s been really phenomenal what we were able to do in such a short period of time and really ramping up because just as a baseline, there isn’t really an infrastructure or really a lot of investment that was made in the health education side of public health or even in healthcare in general. There’s always like an add-on or a tack-on to a budget or maybe it’s really embedded into somebody else’s job role. So just by nature, health education and outreach itself is not a focused area or line item or area of discipline…So, it’s been phenomenal to really have had the resources to do like an outreach team and to recruit community health workers, to recruit educators.*

(Community partner, Health Dept.)

Essential to building this infrastructure was establishing successful partnerships in the form of coalitions with various organizations involved in health education and health equity, to work together to address COVID-19 in BIPOC communities:

*We’re working closely with our FQHC clinics…our hospitals, our academic partners, not just the community partners but other partners that have a role in providing vaccinations, testing, or education because we know that the more we can work together, the better we can be in concerted efforts into our community…I think it’s one of the I would say indicators for the overall COVID work that we have increased the number of partners that are feeling comfortable and working with us over the last 15, 17 months. So definitely, it has been very successful in terms of creating and nurturing those partnerships that are able to reach the pockets we’re focused on.*

(Community partner, Health Dept.)

While initial vaccine distribution took the form of large-scale efforts at stadiums or local healthcare institutions—which presented access issues for under-resourced BIPOC community members, as they required online sign-up, some tech know-how, and reliable transportation—local community and smaller healthcare organizations developed initiatives to bring the vaccine directly to different BIPOC neighborhoods, e.g., to churches and parks, and set up pop-up clinics tailored to community cultural needs and characteristics that used trusted members from inside the community as outreach workers. Neither community residents nor partners reported that access—as a location-related need, in the sense of the vaccine being readily available—was a barrier to receiving the vaccine. However, community partners recognized that access remained a barrier to vaccination for these under-resourced community residents, as it also encompasses—and presupposes—access to the informational and educational resources necessary to understand the vaccine medical information, which is not a given among certain BIPOC communities:

*All these hesitancies are a really big part of how we need to communicate, right? But I think it just goes back to the underlying root causes, the social determinants of health, upstream factors […] If you’ve been involved in public health, none of this is surprising in terms of who has access to information and who’s still lagging behind, who may not have the education – which pockets may have more misinformation. So, I think if we don’t take this opportunity to go more upstream, then I don’t know what it will take.*

(Community partner, Health Dept.)

#### 3.2.4. Interpersonal Factors—Evolving Attitudes towards Vaccination

Both community residents and partners reported that the primary motivation for choosing for or against vaccination was to protect one’s family and oneself. Within community residents’ calculations of weighing risk and uncertainty concerning being vaccinated, some participants indicated that from the perspective of community residents, espousing medical mistrust was the means to safeguard oneself and one’s family:

*It’s an issue of medical mistrust. I mean, that’s a huge part of it. […] So, really, if it’s a vaccine that you want, then there’s a way to get it, but, you know, it might take a step or two on your part to get to a place–and for you to want to take that step, you have to believe that you’re doing the right thing for yourself and for your family*

(Community partner, FQHC)

Similarly, from the perspective of a woman in a Spanish-speaking community, the primary motivation for not being vaccinated was to protect her family and herself, in accordance with her husband’s opinion that not enough was known about the safety of the COVID-19 vaccine:

*I have not had any of the doses of the COVID vaccine because my husband still doesn’t want to and has not allowed me or my daughter […] I think he heard the negative of what has been said about the vaccine, not the positive, he is clinging to that right now, to not do it to take care of me and my daughter. He says, “Let’s wait a little longer to see what happens.”*

(Community resident, Spanish-speaker)

For others, however, protecting their families meant being vaccinated. Community partners reported that they watched community residents’ views on vaccination evolve over time, from initially choosing not to be vaccinated to vaccinating themselves and their family members:

*I think the first reason is their commitment to the family’s health. Some of them said, to protect the grandparent at the house, or to protect the most vulnerable at the house we have to get vaccinated, that is what I’ve heard. The ones getting vaccinated sometimes have not done it for themselves, but to protect the family.*

(Community partner, Spanish-speaking church)

Over time, some community partners and residents who initially were uncertain about being vaccinated reported changing their minds about the vaccine. Various factors contributed to this evolution in their attitudes, including the influence of trusted authorities such as medical, scientific, and religious leaders:

*I didn’t want to get it at first because I wanted to see what the side effects were of people that were getting it. I was that skeptic […] It was Dr. Hotez, but also my physician, Dr. S. She knows everything about me as far as taking care of any ailments or anything I need to have done, so I pretty much trusted her. She says, “It’s better to have it than have not.”*

(Community resident, Gulfton)

Additional factors that influenced a change in perspective on being vaccinated were social norms and networks and the “personal touch,” or hearing personal stories or appeals on the benefits of being vaccinated. Participants’ vaccine status was aligned with those within their immediate social networks; the infrequent exception would be, for example, a lone family member who did get vaccinated because of concerns regarding an underlying medical condition. While community residents noted the value of personal appeals to changing people’s minds about vaccination, community partners saw them as crucial to increasing vaccination within their communities:

*That person is a real person, and that person had an experience. And so based on, “I know that person […] and that person really said this thing was a bad thing,” it affects them. I think it’s the personal story that is probably the greatest influence. I don’t think it’s going to come from a politician. I don’t think it’s going to come from the most famous pop singer or anything like that. I think it’s going to be a personal and absolutely personal relationship that they have.*

(Community partner, English-speaking church)

Several community residents also reported that a conflict of faith and reason also initially led them to question being vaccinated. This experience was more common among Black than among Latino/a/x community residents:

*Because so many of our Christians, Catholic and other, were thinking, “Well, I’ve got the Lord on my side and He’s going to protect me [from COVID].” I heard that over and over and over and that is something–as a Black community, we are faith believers. We are. How we practice our faith can be varied but we are believers and I think it was a struggle for us to understand what our leaders were telling us, “Yes, put your faith in God, but use your brain.” […] some of these televangelists and ministers were saying, “Don’t worry about the virus. It’s going to go away. God’s going to protect you,” and we know that many of that flock died because they were following false prophets.*

(Community resident, English-speaking church)

#### 3.2.5. Individual Factors—Understanding Alternative Health Belief Systems

Many residents who chose not to be vaccinated espoused alternative views on health and the value of vaccinations. Some adopted views concerning the privileging of individual rights over those of the greater community, or espoused views that were misconceptions about vaccine effects (are fatal, cause sterility, infect you with COVID, etc.) and/or conspiracy theories that the vaccine is being used for population control to kill as many vulnerable people as possible:

*I really just think it’s something that the government is adding to us to kill us off honestly. [Laughter] The government just comes up with something new all the time. Like right now, monkeypox. Like it’s always something, cancer, people die from HIV. Why are we not taking shots for that or why are we not curing people from that every day, but you all want us to take two or three different COVID shots, but then you all have got a new strain every month […] The news said the population was crowded in America. Overpopulation […] Well, that’s just in the research in social media.*

(Community resident, Greenspoint 1)

Those espousing conspiracy theories reported heightened fear and feeling unsafe due to increased danger, crime, and a general breakdown of society after the outbreak of COVID-19:

*To survive, the people are on survival tactics right now and they don’t care who they harm or the danger […] in the wake of it [COVID], it’s a sense of desperation out here. It’s a sense of–it’s pandemonium. People are crazy out here right now.*

(Community resident, Greenspoint 2)

Additionally, unvaccinated community residents, unlike those who were vaccinated, also reported highly contradictory beliefs and behaviors. For example, a participant stated her source of COVID-19 and vaccine information was the CDC, yet later she stated:

*It’s [COVID-19] man-made […] It’s really scary because I feel like at one point, they just blew something out in the air then you came by and breathed it. I think they blew 30,000 doses of it because they were dropping in New York, and I’m not even saying it to be scared. New York was unbelievable the way they would just pass it.*

(Community resident, Greenspoint 1)

She expressed fear of COVID-19, believing the infection was a serious threat that killed people, and volunteered for COVID-19 testing at her community church, yet remained unvaccinated because of fearing vaccine effects more than the infection itself:

*Respondent: I think it’s scary.*

*Moderator: You think getting the vaccine for COVID is scary?*

*Respondent: Yes.*

*Moderator: Are you more afraid of the vaccine than COVID itself?*

*Respondent: Yes, absolutely.*

(Community resident, Greenspoint 1)

Others had practical concerns around not being vaccinated—fear of side effects, questioning efficacy of vaccines—given repeated breakthrough infections, as well as espousal of a “natural” and healthy lifestyle, which included historically not getting vaccinated, avoiding use of pharmaceuticals, eating well and exercising, etc. As participants had remained COVID-19-free to date, they did not see the need to be vaccinated. These participants stated that their refusal of vaccination was not based on fear of vaccines:

*I’m not afraid of it. The [family] that passed, they had underlying diseases too like diabetes. So, that’s why I kind of would correlate it with that, but I’m not against the vaccine. I totally agree that it’s people’s choice. Just for me, I don’t really want the vaccine because I don’t know what’s in there, the ingredients and everything, they take longer to test than this particular vaccine, it just was really quick. That’s why. It just changed my mind about my health and other aspects like I work out more, eating healthier. You know what I mean, just being healthy period, but not so much about taking the vaccine.*

(Community resident, Greenspoint 2)

While some community residents reported initially adopting the view of not wanting or needing to be vaccinated due to being young, healthy, and not routinely receiving vaccines, they reported changed attitudes towards vaccination based on concerns for the health of others in their community:

*I’m younger and I was leery about getting it, not fear, but side effects. I don’t take the flu vaccine, every time that I’ve taken a vaccine of that nature, I’ve been down for like 3 weeks. It just really suppresses my immune system. It makes me sick for a long period of time […] I’ll tell you what really encouraged me. Probably made me get it. I had to really think about it and for concern for others. Because everyone that I work with is older than I am. So that really did make me go ahead and get it.*

(Community resident, 3rd Ward)

Personal loss was another individual factor that contributed to changing perspectives on vaccination. Loss led unvaccinated individuals to choose to be vaccinated because psychologically it brought home the gravity of COVID-19, taking the virus from the abstract to the concreteness of experiential grief. In making the threat of the pandemic real, community residents were motivated to protect their remaining loved ones and themselves:

*They are getting the vaccine when they’re scared crapless because somebody in their house got it and died or a friend got it and died. When their friends drop dead or a family member drops dead, that usually puts the “Come to Jesus” to them and then they decide, “Okay, I don’t wanna suffer,” ‘cause they see the suffering, you suffer with COVID. It is not a pleasant experience, you suffer miserably.*

(Community partner, community-based organization)

## 4. Discussion

Vaccine “deliberation” has been used in this study, rather than “hesitancy”; even framing the choice not to be vaccinated as “hesitancy” can arouse moral judgement and the presumption of the rectitude of being vaccinated, rather than evoking interest in understanding why BIPOC community members were questioning vaccination and medical and public health systems. While the choice to be vaccinated or not can have serious public health consequences, adopting a moral judgement stance towards those who are not vaccinated does not further any understanding of the reasons motivating this choice, nor does it promote caring for the health and wellbeing of these community members.

### 4.1. Structural and Community Factors

Research findings identified multilevel factors that interacted dynamically across levels to contribute to vaccine deliberation among under-resourced BIPOC communities, confirming prior research on structural racism as a primary driver of vaccine uptake expressed by participants as merited institutional and medical distrust [22,23,29,39,49,50,51,52,53,54] and as the experience of unyielding “everyday racism” [55]. Participants’ experiences and perspectives regarding COVID-19 are inextricably framed within structural and historical inequities, which shape the responses, power relations, opportunities, conditions, and positions available to BIPOC community members. These long-standing structural inequities in the US—inequitable access to education, housing, healthcare, and employment, as well as the violence of historical racism and disempowerment of BIPOC communities—underlie factors that impacted vaccination uptake, i.e., vaccine *deliberation* and *access*, also noted by other researchers [24,53,56]. Vaccine deliberation and access influenced vaccine uptake and decision-making across multiple levels, conveyed in the different themes and categories expressing participants’ concerns. These include persistent structural racism, a for-profit healthcare system that left participants disenfranchised, politicization of the vaccine, inconsistent national vaccine guidance and communication, media mis/disinformation, educational inequities, underlying medical conditions, fear of vaccine side effects, valid vaccine concerns, and alternative health belief systems.

While access, in terms of easy vaccine availability, was not perceived to be a barrier by most community residents, community partners noted that “access” was contingent upon the SDOHRI and was not simply a location-related issue of vaccine availability. Rather, access was perceived within the contextual framework of individual lives in which vaccination becomes a possibility based on a system of supporting factors. These factors included accurate information on vaccination and the educational resources to understand this medical information, the possibility of missing work to recover from vaccine side effects, having to take time off from work and not be paid to get vaccinated, transportation and digital access, and of possibly paying for the vaccine and being able to provide documents (e.g., ID or medical insurance; because websites for vaccination sign-up requested medical insurance information, it is reasonable to expect that insurance, or alternatively payment, is required). Each of these factors impact community members’ ability to receive the vaccine and together comprise “access.” As echoed by other studies [50,53], participants reported that initial vaccine rollouts requiring online registration failed to reach the most vulnerable members of BIPOC communities, given the requirements of Internet access and an email address. Correcting these digital and transportation inequities requires first acknowledging their existence so as to build programs that can address them [57]. As noted by public and community health partners in this study, this lack of access due to digital, transportation, and education inequities came as no surprise.

Study findings extend previous research by situating the effects of COVID-19 within the daily lives of participants, compounding the intersecting deprivations many were already experiencing—unemployment, food insecurity, unstable housing, and limited access to healthcare—all of which exacerbated psychological stress and heightened the sense of living in survival mode. Food insecurity and increased mental health issues resulting from COVID-19 have been reported [50]. For participants, these competing needs were overwhelming and took priority over protecting themselves from COVID-19. Community members also stated that the outbreak of the COVID-19 pandemic during the widespread uprisings in support of Black Lives Matter served to intensify the perceptions and experiences of feeling unsafe for communities of color. Tackling structural racism within the US will take a committed, multilevel, and multimodal cooperative effort across institutional systems. The establishment of vaccine equity task forces [51,54] at local, state, and national level [58] can serve as a key starting point towards developing programs, policies, structures, and practices [59] for ensuring health equity that can be refocused and sustained to address the different health inequities experienced by BIPOC communities. As public health partners noted in this study, the infusion of resources devoted to addressing COVID-19 allowed for tailored vaccine educational outreach, increased partnerships, and building a coalition of community partners to address the pandemic in BIPOC communities. Increased funding will be necessary to support and sustain local, state, and national initiatives focused on vaccine and overall health equity to be prepared for the next pandemic and other future health crises.

Additionally, the response to the COVID-19 crisis in BIPOC communities required that public health partners build capacity and develop the infrastructure for adequate, culturally tailored health education and outreach programs and partnerships. These partners commended the committed effort devoted to building this capacity that allowed them to effectively respond to the COVID-19 emergency. However, they acknowledged that, had the infrastructure to support these programs already been in place, vaccination outreach efforts among BIPOC communities would have been even more successful. Other researchers have recognized that investment in a structurally competent infrastructure for healthcare education and delivery is imperative to achieving racial justice and health equity in the US healthcare system [23]. Building these partnerships with community members and local organizations invested in health equity also lays the groundwork for developing the organizational capacity and framework to respond to the various health crises affecting BIPOC communities, in addition to COVID-19. Moreover, building such partnerships means that community members can respond to these health crises in accordance with internally identified priorities and terms, thus securing the agency and respect that has been denied to communities of color for far too long and that underlie much of their distrust.

Community partners and residents reported that tailoring vaccination distribution and education campaigns to the needs of individual BIPOC communities was vital to address not only the different cultures, and languages, socioeconomic environments, but also the structural racism that underlies the significant disparities in risk, morbidity, and chronic medical conditions, as well as COVID-19, within these communities [10,29,39,54,60]. Each of the factors related to the community level—media mis/disinformation, underlying medical issues, knowledge of the vaccine, and local tailored vaccine and education campaigns using locals from inside the community to deliver messages—need to be addressed by listening and adapting to community needs. These strategies comprise an equity approach to vaccination, in contrast with an equitable approach. While an equitable approach advocates providing individuals with the same resources and opportunities, equity acknowledges that individuals live with different circumstances, which necessitates distribution of resources and opportunities according to the need to achieve an equitable outcome [61]. Establishing equity has been recognized as primary in addressing the low rates of vaccination within BIPOC communities [20,29]. Our qualitative findings provide insights into the distinct ways in which lack of equity regarding education, intersecting disadvantage, and access—e.g., healthcare, digital access, and transportation—frame participants’ lives and decisions. Community partners were sensitive to listening to community residents’ views on what they needed—i.e., to adopt an attitude of cultural humility, and build partnerships based on bidirectional communication and reciprocity [21,25]. Cultural humility represents the first step in ascending from an equitable to an equity approach to vaccination and considers residents as equal partners in caring for the community, rather than adopting the all-too-familiar attitude of provider and researcher paternalism.

Consistent with other studies [50,54,62], media mis/disinformation was also a crucial concern for community partners and residents, identified by some as the primary barrier to vaccine acceptance, and which required direct intervention to correct community members’ vaccine misconceptions. There is evidence that Black communities have been specifically targeted by various anti-vaccine misinformation or, in this case, disinformation campaigns. NPR reported on an anti-vaccine film entitled *Medical Racism: The New Apartheid*, produced by an anti-vaccine group, Children’s Health Defense, that targets Black Americans with vaccine disinformation through artful contextualization to manipulatively distort COVID-19 vaccination efforts as a continuation of the well-known historical medical racism cases in the US—Dr. Sims, the Tuskegee Syphilis Study, Henrietta Lacks—involving Black Americans [63]. Several researchers, including information scientists [64], media, politics, and public policy experts [65], and communication specialists [66], have reported on the targeting of Black communities during the ongoing COVID-19 pandemic, through anti-vaccine campaigns. Interventions implemented across the US have focused on addressing misinformation and building vaccine confidence using tailored, multimodal approaches led by trusted messengers, including online initiatives such as interactive webinars, public education campaigns, and town halls live-streamed on social media, as well as in-person and grassroots initiatives employing culturally informed community outreach, which have been effective [51]. This targeting of Black communities by anti-vaccine campaigns could also be a factor contributing to community residents’ reports of confusion and contradictory beliefs concerning COVID-19 and the vaccination.

### 4.2. Interpersonal and Individual Factors

Data collection for this study covered a broad span of time—lasting roughly a year, from August 2021 to September 2022—as well as changes in the trajectory of the pandemic, from initial vaccine rollouts to outbreaks of different SARS-CoV-2 variants. Notably, community partners and residents reported evolving attitudes towards vaccination, moving from initial distrust to vaccine acceptance. The primary reasons for deciding on vaccination included being convinced of its safety, taking the advice of a trusted authority, and being touched by the personal suffering of family or community members due to COVID-19. Unfortunately, it took the loss of loved ones to COVID-19 to change many participants’ views on vaccination to protect themselves and their families. Many participants waited to see how others reacted before deciding to be vaccinated, as also cited by other researchers [53]. Likewise, trusted authorities, such as medical, scientific, and religious leaders, were effective in changing community residents’ views on being vaccinated [67]. Several community partners also noted that personal stories or testimonies by community residents on how their vaccination views had evolved from vaccine deliberation to acceptance had been very effective in convincing others to do the same. Results of a survey study examining changes in vaccine acceptance between Black and White adults in the US found that Black participants came to accept vaccines more quickly than White counterparts [24]. Notably, these findings support that Black Americans are amenable to changing from initial vaccine mistrust once they are confident of the protective benefits of vaccination for their families and communities. Additionally, several Black community residents related initially experiencing a conflict between faith and reason, feeling that as people of faith, God would protect them from being infected with SARS-CoV-2. Being aware of the prevalence of this view among Black faith communities, religious leaders directly spoke to these concerns, guiding their congregants in making a decision that respected and addressed their spiritual conflicts about vaccination through seeing that there is no conflict between believing scientific information on the effectiveness of vaccines and having faith in God. Unfortunately, participants also noted that in some Black faith communities, religious leaders were continuing to tell congregants not to worry about being vaccinated against COVID-19, because as people of faith they were protected against the virus. This finding on the role that faith plays in being vaccinated adds to our understanding of vaccine barriers among Black communities and emphasizes the need to work with religious leaders as partners in vaccination efforts, given the position of authority that faith leaders often occupy within their communities [39,68].

Another reason cited for not being vaccinated was fear of vaccine side effects [54,69]. Participants reported a fear of unsubstantiated side effects gleaned from social media—i.e., death, contracting COVID-19, paralysis, and sterility—as a vaccine barrier. A novel finding was that several participants in this latter group stated they were more afraid of being vaccinated than of contracting the virus. The presence of these heightened fears, as well as the contradictory beliefs espoused by some unvaccinated community residents, speak to a level of dread and alarm that is seemingly unfounded. These fears point to a vague, unnamed, and pervasive apprehension expressed by participants, as evidenced by narratives of increased crime, danger, and the breakdown of the social fabric due to the state of desperation—e.g., high unemployment, food scarcity, and high COVID-19 rates—BIPOC community members were facing due to COVID-19. Participants’ expression of this pervasive apprehension suggests the degree to which they were perceiving their lives as precarious and vulnerable; that they are, as one participant reported, living in “unsafe places,” attributable to the realities of persistent structural racism.

Several residents who chose not to be vaccinated espoused alternative views on health and the value of vaccinations. Some of these views were based on unproven misconceptions of the effects of the vaccines and/or conspiracy theories; e.g., that the vaccine is being used for population control to kill as many vulnerable people as possible. Others, however, were based on concerns related to participants’ observations or general approaches to health—questioning vaccine efficacy due to repeated breakthrough infections, adopting of a “natural” and healthy lifestyle (i.e., historically do not get vaccinated and avoid consumption of pharmaceuticals), eating well and exercising, etc.—through which participants had remained COVID-19-free to date so they did not see the need to be vaccinated. For these people—who, within our sample, were all Black American adults—receiving vaccinations of any sort did not fit with their worldview, and the potential costs outweighed the potential benefits. This latter group did not express fear of the vaccine, as did those in the former group, but stated they simply found vaccines to be unnecessary; that they felt they could live safely without them by taking care of their health through natural means, e.g., maintaining healthy diets and exercising. This finding of adopting alternative preventive measures that aligned with natural health was also cited by other studies [53,67].

Given the for-profit US healthcare system, the adoption of an alternate health system makes sense, especially among under-resourced people of color, who have valid reasons to distrust US medical care based on ongoing structural racism. These individuals cannot afford to engage in the health promotion/health prevention model that is framed within the structures of a predominantly White middle-class life [70]—i.e., medical insurance, employment that provides medical insurance coverage, regular medical checkups (covered by insurance), and paid time off to attend medical appointments and preventive care—none of which is available to many vulnerable BIPOC community members. Adopting an alternative, more “naturalistic” health system, whereby these community members essentially attend to their own healthcare as much as possible, is reasonable, because (1) systemic racism has kept these communities out of the middle-class health promotion/prevention system, that can be linked to power and privilege; (2) this is the only affordable healthcare that is available; and (3) given merited limited contact with the healthcare system, this places healthcare largely within individual control. Although participants who espoused alternative health beliefs did not express fear of the vaccine, they did report institutional distrust, particularly in regard to the pharmaceutical industry, bringing this theme back again to underlying structural racism.

### 4.3. Summary of Recommendations

Addressing structural racism is critical to the effort to promote vaccine acceptance within BIPOC communities [39,51,54]. Crucial to this effort is adopting an empathetic perspective; it is imperative to work under the assumption that most people, most of the time make choices that are sensible to them, given the conditions that constrain their lives. As a community resident, who had been vaccinated, asked: “*So, considering everything we have gone through* [with COVID] *and having a way to prevent it* [vaccination]*, who would decide not to make the decision to prevent it and protect oneself?”* Although it may not be apparent from the outside, the primary motivation for participants who chose not to be vaccinated was to protect their families and themselves. Given historic antecedents in the US, mistrust of government and medical institutions is more than merited for BIPOC community members, and it has been necessary to protect the very lives of these communities.

The following recommendations summarize and extend suggestions made by community partners and residents on how best to increase vaccine acceptance among BIPOC communities by equipping community members with the tools that empower and facilitate informed decision-making about vaccination. Recommendations are informed by and incorporate suggestions garnered from a review of the literature [51,54]. To address the racial and health inequities and disempowerment of BIPOC communities, implementation researchers advocate adopting explicitly racial equity- and anti-racism-based approaches to ensure that existing inequities are not perpetuated or exacerbated by traditional frameworks, methods, strategies, and interventions that do not consciously account for the effects of structural racism [31,71]. Any efforts specifically tailored to these heterogenous communities must be explicitly focused on equity and adopt an anti-racist lens to elicit members’ engagement, empowerment, and agency as collaborators in addressing this pandemic within their respective communities. Given the heterogeneity of BIPOC communities, it is crucial that public health and community healthcare organizations continue their efforts to: (1) listen to community members’ needs and concerns, acknowledge uncertainties on vaccines and institutional distrust, and hear members’ healthcare priorities and interests to inform initiatives and practices built on locally gathered data; (2) address mis/disinformation to dispel myths through culturally informed messaging and communication based on listening and tailoring messaging to community concerns, and to ensure consistent messaging, delivered by trusted community leaders (representatives of faith-based organizations and community-health organizations), either in-person or via virtual town halls, public education campaigns, and discussions; (3) take interventions and vaccine distribution to where residents live (e.g., pop-up clinics, recreational parks, grocery stores, community centers, and churches) delivered by trusted community members and partners, tailoring education and communication campaigns to the needs of these distinct communities; (4) establish vaccine equity task forces to develop sustainable structures, policies, programs, and practices to address the myriad structural issues underlying vaccine and health inequities within communities of color; and (5) continue to invest in a structurally competent infrastructure for healthcare education and delivery, essential for responding effectively to the ongoing healthcare and other emergency crises that impact BIPOC communities to achieve racial justice and health equity in the US. While there have been few interventions focused on increasing vaccine uptake among BIPOC communities, Abdul-Mutakabbir et al. [39] report on the effective implementation of a rapid community–academic model for promoting COVID-19 vaccine equity in BIPOC communities, which shows the importance of tailoring vaccine education and delivery efforts by employing racially concordant healthcare professionals and outreach personnel. Moreover, it is necessary to provide greater funding and resources to local community organizations and leaders, as well as local public health departments through national, state, or local sources to continue to develop and sustain their efforts to achieve vaccine and health equity [51].

### 4.4. Study Limitations and Strengths

There are a few study limitations to note. Focus group participants were recruited from local community organizations with whom researchers had established partnerships, and with community residents who were members of these organizations. As many of these organizations were partners in the effort to disseminate COVID-19 vaccines within BIPOC communities, the sample in this study was unique and may not be representative of members of other BIPOC communities. A strength of this study, however, is that it captures and presents some of the diverse views of vaccinated and non-vaccinated community members in Houston, Texas, the fourth-largest city in the US. Given COVID-19 concerns, several focus groups were conducted virtually which, given access and connectivity issues, may have limited the participation of the most vulnerable in these communities. Future studies with BIPOC communities need to consider the limitations imposed by digital inequities and find alternative means of working with these participants. While culturally matched members of the research team were present for all focus groups for this study, virtual and in-person, the focus groups themselves were conducted by a White-presenting, Spanish-speaking Latina. To facilitate greater rapport and comfort of participants, future studies should use culturally matched interviewers to create an environment where BIPOC community members can feel safe to share their views openly.

As the data collection for this study encompassed a broad timespan of August 2021–September 2022, participants’ experiences across different surges and variants of SARS-CoV-2 and their evolving attitudes towards the pandemic and vaccination were captured. Focus groups were conducted after COVID-19 vaccines became available, thus capturing participants’ behaviors regarding vaccination rather than their projected intentions, as reported by other studies [53,62]. The current work also presents the views of both community partners and community residents, providing insights into the different challenges and successes that partners experienced with vaccine outreach as well as the experiences and perspectives of community residents regarding vaccine decision-making, and the barriers and facilitators to vaccine access. Together, these different perspectives provide a more comprehensive view of the issues and concerns that BIPOC communities are facing in regard to COVID-19 vaccination that will be used to inform the development of a culturally responsive vaccine education campaign in BIPOC communities, focused on health equity.

## 5. Conclusions

Findings point to the crucial need to understand and address the core issue responsible for the interrelated factors that together yield health and vaccine inequities: structural racism. The work to build trust with BIPOC communities is essential and entails adopting cultural humility to be capable of listening to community members and empathizing to view the experience of COVID-19 from inside the perspective of community members to develop partnerships founded on reciprocal respect. While attitudes and behaviors towards vaccination evolved over time among our participants, much work remains to be done to earn the trust of these heterogenous populations, as—contrary to public perception—COVID-19 continues to be a danger. Another challenge involves understanding and responding to the healthcare needs of individuals who have espoused alternative health beliefs and systems. To improve the health outcomes of under-resourced BIPOC populations, we must first understand the health beliefs, perspectives, goals, barriers, and priorities of community members if we wish to change the mainstream system of health prevention/promotion in order to meet these members’ needs, as well as advance healthcare utilization and vaccine confidence.

## Figures and Tables

**Table 1:** Sample characteristics of focus group participants from online survey by phase in Houston, Texas (*n* = 55).

Characteristics	Phase 1 Partners (*n* = 19) ^§^	Phase 2 Residents (*n* = 36) ^§^
	Mean (SD)/% [*n*]	
Age	47.32 (11.46)	49.86 (18.10)
Sex		
Male	42.11 (8)	32.35 (11)
Female	57.89 (11)	67.65 (23)
Ethnicity/Race		
Asian	10.53 (2)	2.94 (1)
Black	57.89 (11)	79.41 (27)
Latino/a/x	21.05 (4)	11.76 (4)
White	5.26 (1)	0 (0)
Biracial/Multiracial	5.26 (1)	5.88 (2)
Education		
Some high school	0 (0)	8.33 (3)
High school diploma or GED	0 (0)	27.78 (10)
Some college or 2-year degree	21.05 (4)	33.33 (12)
4-year college graduate	21.05 (4)	19.44 (7)
Some school beyond college	5.26 (1)	2.78 (1)
Graduate or professional degree	52.63 (10)	8.33 (3)
Total Household Income		
Under USD 29,999	10.53 (2)	38.89 (14)
USD 30,000–59,999	5.26 (1)	22.22 (8)
USD 60,000–89,999	31.58 (6)	5.56 (2)
USD 90,000–119,999	21.05 (4)	11.11 (4)
USD 120,000–149,999	5.26 (1)	5.56 (2)
USD 150,000–249,999	15.79 (3)	0 (0)
Over USD 250,000	5.26 (1)	0 (0)
I don’t know	5.26 (1)	16.67 (6)
Employment Status		
Working full-time	84.21 (16)	31.43 (11)
Working part-time	0 (0)	25.71 (9)
Unemployed and looking for a job	0 (0)	28.57 (10)
Retired	10.53 (2)	8.57 (3)
Disabled	0 (0)	2.86 (1)
Enrolled in school/college/university	5.26 (1)	2.86 (1)
Insurance		
Yes	84.21 (16)	69.44 (25)
No	15.79 (3)	30.56 (11)
Vaccine Status		
Vaccinated	87.50 (14)	44.83 (13)
Not vaccinated	12.50 (2)	55.17 (16)

Notes: ^§^ Of the 22 partners from Phase 1, 19 completed online surveys; of the 57 residents from Phase 2, 36 submitted their online surveys.

## Data Availability

Data are not publicly available due to privacy restrictions. The data that support the findings of this study are available from the senior author, E.M.O., upon reasonable request.
